# Physiological impact on layer chickens fed corn distiller’s dried grains with solubles naturally contaminated with deoxynivalenol

**DOI:** 10.5713/ajas.19.0199

**Published:** 2019-07-01

**Authors:** Samiru Sudharaka Wickramasuriya, Shemil Priyan Macelline, Eunjoo Kim, Hyun Min Cho, Taeg Kyun Shin, Young Joo Yi, Dinesh D. Jayasena, Sung-Dae Lee, Hyun Jung Jung, Jung Min Heo

**Affiliations:** 1 Department of Animal Science and Biotechnology, Chungnam National University, Daejeon 34134, Korea; 2 Department of Animal Science, Uva Wellassa University, Badulla 90000, Sri Lanka; 3 Division of Biotechnology, Chonbuk National University, Iksan 54596, Korea; 4 National Institute of Animal Science, RDA, Cheonan 31002, Korea

**Keywords:** Corn Distillers Dried Grains with Solubles, Deoxynivalenol, Egg, Laying Hens

## Abstract

**Objective:**

An experiment was conducted to investigate the response of laying hens fed corn distiller’s dried grains with solubles (DDGS) that are naturally contaminated with deoxynivalenol (DON).

**Methods:**

One hundred and sixty 52-week-old Lohmann Brown Lite hens were randomly allotted to five dietary treatments with 8 replicates per treatment. The dietary treatments were formulated to provide a range of corn DDGS contaminated with DON from 0% to 20% (i.e., 5% scale of increment). All laying hens were subjected to the same management practices in a controlled environment. Body weight, feed intake and egg production were measured biweekly for the entire 8-week experiment. The egg quality was measured biweekly for 8 weeks. On weeks 4 and 8, visceral organ weights, blood metabolites, intestinal morphology, and blood cytokine concentrations were measured.

**Results:**

The inclusion of corn DDGS contaminated with DON in the diet did not alter (p> 0.05) the body weight, feed intake, hen-day egg production, egg mass and feed efficiency of the laying hens. No difference was found (p>0.05) in the egg quality of hens that were fed the dietary treatments. Furthermore, hens that were fed a diet containing corn DDGS contaminated with DON showed no change (p>0.05) in the visceral organ weights, the blood metabolites, and the cytokine concentrations. The crypt depth increased (p<0.05) as the amount of corn DDGS contaminated with DON increased. Proportionately, the villus height to crypt depth ratio of the laying hens decreased (p<0.05) with the increasing level of corn DDGS contaminated with DON in the diet.

**Conclusion:**

The inclusion of corn DDGS contaminated with DON up to 20% in layer diets did not cause changes in egg production performance and egg quality, which indicates that DON is less toxic at the concentration of 1.00 mg DON/kg.

## INTRODUCTION

Mycotoxins are becoming more abundant in the cereals, and their products cause a significant negative influence on the animal feed industry. Total mycotoxin contamination accounted for 25% to 50% in world crop production [1]. Deoxynivalenol (DON) is a naturally existing mycotoxin that belongs to the trichothecene family. *Fusarium graminearum* and *Fusarium culmorum* are the major fungi that are responsible for producing the DON mycotoxin [2]. The fundamental mode of action of trichothecenes is to block the peptidyl transferase of 60S ribosomal subunits and thereby inhibit protein synthesis [2–4].

There are mounting concerns about the prominent effect of DON on performance and immune responses in monogastric animals [5]. However, many researchers have reported that layers seem to be tolerant to DON, and no responses have been demonstrated in the growth performance, egg production and egg fertility [6,7]. Nevertheless, contradictory adverse influences were observed with the performance index and immune responses of laying hens [8]. The different observations can be attributed to the degree of DON contamination in feed and the synergetic effect of other mycotoxins in layer diets. However, to our knowledge, limited information is known about the impact of layer fed corn distiller’s dried grains with solubles (DDGS) that is naturally contaminated with DON.

Thus, the objective of the current study was to determine the layer response to dose-dependent inclusion of corn DDGS that are naturally contaminated with DON into the layer diet, with a special focus on production performance, egg quality, blood metabolites, organ weights, gut morphology and cytokine concentrations of laying hens.

The present study tested the hypothesis that higher inclusion of corn DDGS that are naturally contaminated with DON in layer diets will decrease the production performance and egg quality, as well as gut health.

## MATERIALS AND METHODS

### Animal care

The animal protocol for the current research was reviewed and approved by the Chungnam National University Animal Ethics Committee (Protocol No. CNU-00980).

### Birds and housing

One hundred and sixty 52-week-old Lohmann Brown Lite laying hens were obtained from the commercial farm (75-39, Juraebonjuk-ro 590beon-gil, Yul-myeon, Icheon-si, Gyeonggi-do, Republic of Korea). Upon the arrival laying hens were introduced into the experimental facility and let acclimatized for the period of two weeks. Four laying hens were housed in each battery cage equipped with a feeder and two nipple drinkers. Same management practices were subjected to all laying hens in an environmentally controlled room maintained at 26°C±1°C. Throughout the experiment, 16 hours of lighting and 8 hours of the dark was maintained.

### Experimental design, diets and treatments

At the beginning of the experiment, initial body weight of the laying hens was recorded and allocated into 1 of 5 dietary treatments with 8 replicate cages per dietary treatment (4 laying hens in one cage) in a completely randomized design.

Five dietary treatments were formulated to contained corn DDGS that are naturally contaminated with DON from 0% to 20% (as fed basis) in 5% increment in dose dependent manner. Natural deoxynivalenol in corn DDGS was analyzed by HPLC methods (Danicke et al [8]) and found to contained 5 mg DON/kg. Accordingly, calculated DON levels of five different diets were 0, 0.25, 0.50, 0.75, and 1.00 mg DON/kg. All the formulated diets consisted of the same energy and protein levels to meet or exceed NRC [9] specifications for layers (Table 1). Laying hens were offered the respective experimental diet on an *ad libitum* basis for the period of study and fresh water was available at all times.

### Data collection

#### Measurements

Pen basis body weight and feed intake were measured every 2 weeks interval from the beginning of the experiment. Feed intake was measured as the feed dessaperance from the feeder.

Pen basis egg production was recorded and collected throughout the experiment. Hen-day egg production, egg mass and feed efficiency were calculated according to methods described by Ali et al [10] and Singh et al [11]. Eggs were labeled and collected separately on weeks 2, 4, 6, and 8 for egg quality analysis.

#### Egg quality parameters

Collected all eggs were weighed using analytical balance (OHAUS explorer E12140, Ohaus Corp., Florham Park, NJ, USA) and recorded separately based on the pen. Total of 320 eggs (16 eggs from each treatment at a time) were randomly selected from the total egg collection for internal and external quality analysis. Egg quality measurements were performed as the methods described by Wickramasuriya et al [12].

The egg width and length were measured using a digital caliper (MITUTOYO 530-312, Mitutoyo, Japan). Egg shape index was calculated based on egg length and egg width using the following equation according to Jayasena et al [13].

Egg shape index=(width of egg/length of egg)×100

The static compression eggshell strength was measured with the aid of a texture analyzer stable microsystem (TA.XT Plus Texture Analyzer, Texture Technologies Corp., Scarsdale, NY, USA). Shell color reflectometer (TSS QCR, Technical Services and Supplies, York, UK) was used to measure the eggshell color. Thick albumen height and the Haugh unit of each egg was measured using an automated Haugh unit system (TSS QCD, Technical Services and Supplies, UK). Yolk color was measured by utilizing yolk colorimeter (TSS QCC, Technical Services and Supplies, UK) that initially calibrated with DSM color fan.

### Sample collection

At the end of week 4 and 8 of the experiment, one bird from each replicate cage (8 laying hens from each treatment) was selected and recorded the live weight before the samples collection. Blood samples were collected from the wing vein into spray-coated K2 EDTA vacutainer tubes (BD Vacutainer, Franklin Lakes, NJ, USA) and BD Vacutainer SST (BD Biosciences, Franklin Lakes, NJ, USA) tubes contain polymer gel for serum separation. Collected blood samples were transferred immediately for further processing.

Thereafter, laying hens were sacrificed by cutting the carotid artery and jugular vein followed bleeding as described previously by Mota-Rojas et al [14]. To collect the jejunum samples, the abdominal cavity was opened and exposed the gastrointestinal tract. The Jejunum was defined as midway between the point of entry of the bile duct of duodenum and Meckel’s diverticulum [15] Approximately 3 cm of the jejunum tissue sample from the distal jejunum was excised, immediately flushed with ice-cold phosphate-buffered saline (pH 7.4) and fixed with 10% neutral-buffered formalin solution for determination of mucosal morphology.

Liver, spleen, ovary and kidney were removed from each bird and weighed. All these organ weights were expressed as proportionately to the live body weight of the respective individual bird.

### Sample preparation and laboratory analyses

Blood samples were centrifuged (Micro 12, Hanil Science Co., Ltd., Korea) at 3,000×g for 10 min at 4°C and separated the serum. Separated serum samples were stored in Eppendorf tubes at −80°C (UniFreez U 400, DAIHAN Scientific Co., Ltd, Wonju, Korea) for later analysis. Albumin, blood urea nitrogen (BUN), total protein, globulin and blood enzymes, including alanine transaminase (ALT), aspartate aminotransferase (AST), and gamma-glutamyl transferase (GGT) levels were assayed in the serum samples by an Automatic Biochemistry Analyzer 7180 (HITACHI, Tokyo, Japan) using commercially available kit.

Mononuclear cells were separated from the whole blood using SepMate (STEMCELL Technologies Inc. Vancouver, BC, V6A 1B6, Canada) tube with the aid of Lymphoprep (Axis-Shield PoC AS, Oslo, Norway.) solution according to the method describe Stone et al [16]. Harvested peripheral blood mononuclear cells were then cleaned, pelleted and stored at −80°C (UniFreez U 400, Daihan Scientific Co., Ltd, Korea) for later analysis.

Total RNA was extracted from lymphocytes using RNAiso plus (Takara, Otsu, Japan). The RNA concentration was confirmed by BioSpec-nano (Shimadzu, Kyoto, Japan). After 500 ng of RNA was heated at 65°C for 5 min, cDNA was synthesized using ReverTra Ace qPCR RT kit (Toyobo, Osaka, Japan). The relative gene expressions of interleukin one beta (IL-1β), interleukin six (IL-6), tumor necrosis factor alpha (TNF-α), and interferon gamma were measured by quantitative real-time polymerase chain reaction (PCR) (StepOnePlus real-time system, ThermoFisher Scientific, Waltham, MA, USA) with SYBR green PCR reagent (TOPreal qPCR 2X Premix, enzynomics, Daejeon, Korea) and normalized to the level of b-actin as a reference gene. The primers sequences for those genes were shown in Table 2.

Jejunum samples were subjected to the sample preparation process. Ring-shape and longitudinal shape tissue samples were excised and dehydrated followed by impregnated in paraffin wax. From each of these, 6 transverse sections (4 to 6 μm) were cut using microtome, stained with hematoxylin and eosin, and mounted on glass slides. Histological indices were measured using NIS-Elements Viewer software (Version: 4.20; NIS Elements, Nikon, Melville, NY, USA) with an inverted microscope (Eclipse TE2000, Nikon Instruments Inc., Melville, NY, USA) using a calibrated eyepiece graticule at 4× magnification. The height and the width of 10 well-oriented villi and their associated crypts were measured from each slide according to Özdoğan et al [17]. The villus height to crypt depth ratio (V:C) was calculated.

### Statistical analysis

Data were analyzed as a completely randomized design, using the general linear model procedure of one-way analysis of variance in SPSS software (Version 24; IBM SPSS 2016). The orthogonal polynomial contrasts were used to determine the linear and quadratic effects of increasing concentrations of corn DDGS naturally contaminated with DON into the layer diet. The pen was used as the experimental unit for growth performance and egg production performance. Selected individual laying hens were considered as experimental units for the blood metabolites, gut morphology, and visceral organ weights. Diet effect (corn DDGS level) was the single factor considered for analysis. When treatment effect was significant (p<0.05), means were separated using Tukey’s multiple range test procedures of SPSS software (Version 24; IBM SPSS 2016).

## RESULTS

The results of the body weights and feed intake of the layers fed different level of corn DDGS contaminated with DON are presented in Table 3. There was no effect (p>0.05) of the addition of corn DDGS contaminated with DON into the diet on the body weight of the laying hens during the 8-week experimental period. Similarly, feed intake did not differ (p>0.05) among the treatments up to week 6 of the experiment. Following week 8, laying hens fed with a diet containing 15% contaminated corn DDGS had a higher (p<0.05) feed intake compared with those fed the 0% contaminated corn DDGS diet.

Egg production performance and feed efficiency of the layers fed diets with different levels of corn DDGS contaminated with DON appear in Table 4. No differences (p>0.05) among the dietary treatments were observed for hen-day egg production, egg mass and feed efficiency of the laying hens. Similarly, the inclusion of corn DDGS contaminated with DON into the layer diets up to 20% did not affect (p>0.05) the internal and external quality measures of the egg (Table 5).

Differences in the proportionate liver, spleen, ovary and kidney weights were not observed (p>0.05) among laying hens that were fed different dietary treatments at weeks 4 and week 8 of the experiment (Table 6). Moreover, feeding corn DDGS contaminated with DON did not affect (p>0.05) the blood metabolites of the laying hens (Table 7).

The results of the intestinal architecture analysis of the layers fed different level of corn DDGS contaminated with DON are presented in Table 8. The increasing level of corn DDGS contaminated with DON in the diet had a higher (p<0.05) crypt depth associated with a lower (p<0.05) V:C of the laying hens in weeks 4 and 8 of the experiment. Nevertheless, no alternations (p>0.05) were observed among treatments for the villus height and villus width during the experimental period.

Moreover, no effect (p>0.05) was observed in the cytokine expression of the laying hens in week 4 for corn DDGS contaminated with DON-added diet (Table 9). However, in week 8, with the increasing level of corn DDGS contaminated with DON suppressed the IL-1β cytokine level in the blood.

## DISCUSSION

The wide range of naturally occurring mycotoxins in grains, including oats, wheat, corn, barley and sorghum, are critically harmful and greatly affect the performance and productivity of poultry [18]. Animals fed grain contaminated with DON had detectable toxins in the end products [19]. Although previous poultry studies showed inconsistent results of DON, layer chickens were considered to be more resistant to DON and had fewer impacts compared to other production animals [18,20].

In the present study, laying hens fed corn DDGS contaminated with DON up to 20% (1 mg DON/kg) had no effect on the body weight, egg production and feed efficiency. In agreement, Sypecka et al [21] did not observe any adverse effects on the egg production performance of laying hens that were fed a diet contaminated with 10 mg/kg DON. Similarly, the feed intake was also not influenced by the inclusion of corn DDGS contaminated with DON into the diet up to week 6 of this experiment. On week 8, laying hens fed with 15% corn DDGS contaminated with the DON-added diet showed a higher feed intake compared to that of laying hens fed a diet without adding corn DDGS contaminated with DON. Observations of this pronounced feed intake difference after week 6 suggestied that DON may possess an effect after prolonged exposure. With a similar notion, Lucke et al [20] noted the DON effects expression did not depend solely on the dose but also on the exposure period of the laying hens. Nevertheless, the confounding results of the feed intake in week 8 were contrary to the previous reports, which reported that DON to reduced feed intake by increasing the satiety [20]. However, the reason for this opposition to the feed intake results in this study is unknown and unclear.

The effects of the egg quality parameters of the laying hens fed a diet contaminated with DON were reported in previous studies [22]. Higher egg weight and lower eggshell proportion and eggshell weight were noticed in the laying hens fed a diet that was contaminated with DON in different studies [6,8,22]. Nevertheless, responses of the egg quality parameters in the present study were not affected by the corn DDGS contaminated with DON addition into the layer diets. An explanation for these observed differences in the egg quality parameters in previous studies and in our study could be the low contamination level of DON (below 1 mg DON/kg) in our study compared to that of other experimental diets. In addition, these differences could be explained by the different DON sensitivities among different layer breeds, as was observed by Chen et al [22].

Organ atrophy or swelling-related weight abnormalities reflected the inflammatory states or possible tissue damage caused by toxins [20]. In a previous study, Awad et al [23] reported that DON reduced relative kidney weight, which suggested that DON damaged the kidney cells. With this in mind, the liver, spleen, ovary and kidney weight of the laying hens in this study were measured and were expressed proportionately to the live body weight of the respective birds. However, different dietary treatments had no effect on the relative liver, spleen, ovary, kidney weights of the laying hens in the present study. Consistent with our results, previous studies found that liver, spleen, bursa and heart weights of the chickens and turkeys were not affected by DON contaminations in the diet [4,7,8]. Perhaps the observation can be ascribed to the fact that chickens are less sensitive to DON toxins. No difference was observed in the relative organ weights in this study, which further supported the blood metabolites results of the study.

In the present study, the addition of corn DDGS contaminated with DON into the layer diet up to 20% did not affect the blood albumin, BUN, total protein and blood globulin of the laying hens. Moreover, laying hens fed with added corn DDGS contaminated with a DON diet did not influence the blood enzyme levels of ALT, AST, and GGT. All blood biochemical parameters towards the protein metabolism were analyzed in the present study, in view of the protein synthesis inhibitor potential of DON [22,24]. Nevertheless, no differences in blood parameters were observed, which may be attributed to the lower DON concentrations (1.00 mg/kg) in the experimental diet. Chen et al [22] observed no abnormalities of blood AST and ALT levels as functional indicators of the liver and no liver weight changes in the slow-growing black-feathered Taiwan country chickens that were fed a diet contaminated by DON up to 10 mg/kg. In support of this notion, no liver weight differences, along with similar blood metabolites, were observed among treatments of this study.

As evidenced, DON altered the small intestinal morphology of the chickens, specifically in the duodenum and jejunum with thinner and shorter villi together with irregular crypts [7]. Similarly, corn DDGS contaminated with DON-fed laying hens in the present study showed a higher crypt depth associated with a lower V:C at weeks 4 and 8 of the experiment. Interestingly, between 10%–20% inclusion of corn DDGS contaminated with DON in the diet observed a similar crypt depth and villus to crypt depth ratio of the laying hens. The higher crypt depth commensurate with lower villus height that was observed could be ascribed to the fact that the DON toxin affects the intestinal morphology and functions. Nonetheless, the jejunum villus height and villus width were not affected by the addition of the DON contaminated corn DDS to the diets of laying hens.

Awad et al [7] reviewed the effect of DON on cytokine gene expression, although information on cytokine expression in chickens when fed naturally contaminated grains was lacking. Similar to most of the other measured indices in this study, cytokine expressions levels also did not show any differences among the treatments in week 4. In mice, DON induced the mRNAs for the proinflammatory cytokines when fed with a higher dosage (above 5 to 25 mg/kg body weight), and the effect was nonsignificant for lower doses [25]. Moreover, it was reported that feeding grains naturally contaminated by up to 12 mg of DON/kg was not immunotoxic to poultry [7]. Feeding DON for a long time resulted in lower TNF-α in plasma, which indicated that DON could affect immune function, therefore, leading to a higher infectious disease susceptibility in chickens [18]. Even though the TNF-α level of this study was not affected week 8, the observed lower IL-1β in plasma may follow the same mechanism as TNF-α. This observation may also be related to the observed higher cell turnover (lower V:C ratio) in the small intestine.

In conclusion, hens that were fed a diet containing corn DDGS contaminated with DON showed no changes in egg production performance and the egg quality indicate that DON is less toxic at the concentration of 1.00 mg DON/kg. However, further investigation to determine the breakthrough DON dosage that affect layer performance and egg quality is warranted.

## Figures and Tables

**Table 1:** Composition of experimental diets (% as fed)

Items	Treatment (contaminated corn DDGS level)				

	DDGS-00	DDGS-05	DDGS-10	DDGS-15	DDGS-20
Corn	56.17	54.40	52.63	50.85	49.08
Corn DDGS	-	5.00	10.00	15.00	20.00
Wheat bran	3.55	2.66	1.78	0.89	-
Soybean meal, 48%	26.35	23.95	21.55	19.15	16.75
Vegetable oil	2.00	2.08	2.17	2.25	2.33
Limestone	9.20	9.21	9.23	9.24	9.25
Dicalcium phosphate	1.95	1.86	1.78	1.69	1.60
Iodized Salt	0.30	0.30	0.30	0.30	0.30
Vit-Min premix1)	0.30	0.30	0.30	0.30	0.30
Lysine-HCl		0.06	0.12	0.17	0.23
DL-methionine	0.18	0.18	0.17	0.17	0.16
Calculated composition					
ME (kcal/kg)	2,800	2,800	2,800	2,800	2,800
Crude protein (%)	17.9	17.9	17.9	17.9	17.9
Calcium (%)	4.1	4.1	4.1	4.1	4.1
Available phosphorus (%)	0.46	0.46	0.46	0.46	0.46
Total lysine (%)	0.97	0.97	0.97	0.97	0.97
Total methionine (%)	0.46	0.46	0.46	0.46	0.46
Total Met+Cys (%)	0.77	0.77	0.77	0.76	0.76
Total threonine (%)	0.68	0.67	0.66	0.66	0.65
Total isoleucine (%)	0.74	0.73	0.72	0.71	0.70
Total tryptophan (%)	0.21	0.20	0.20	0.19	0.18
Total valine (%)	0.84	0.84	0.84	0.83	0.83
Total arginine (%)	1.14	1.09	1.04	0.99	0.94
DON level2) (mg/kg)	0.00	0.25	0.50	0.75	1.00

DDGS, distiller’s dried grains with solubles; ME, metabolizable energy.

1) Supplied per kilogram of total diets: Fe (FeSO_4_·H_2_O), 80 mg; Zn (ZnSO_4_·H_2_O), 80 mg; Mn (MnSO_4_·H_2_O), 80 mg; Co (CoSO_4_·H_2_O), 0.5 mg; Cu (CuSO_4_·H_2_O), 10 mg; Se (Na_2_SeO_3_), 0.2 mg; I, (Ca(IO_3_)·2H_2_O) 0.9 mg; vitamin A, 24,000 IU; vitamin D_3_, 6,000 IU; vitamin E, 30 IU; vitamin K, 4 mg; thiamin, 4 mg; riboflavin, 12 mg; pyridoxine, 4 mg; folacine, 2 mg; biotin, 0.03 mg; vitamin B_8_, 0.06 mg; niacin, 90 mg; pantothenic acid, 30 mg.

2) Values are calculated based on the corn DDGS inclusion level.

**Table 2:** The Primers for quantitative real-time polymerase chain reaction

Cytokines		Primer sequence	Gene Bank accession
IL-1β	Forward	5′-ATTACCGTCCCGTTGCTTT-3′	NM_204524
	Reverse	5′-AGTCACAATAAATACCTCCACCC-3′	
IL-6	Forward	5′-AAATCCCTCCTCGCCAATC-3′	NM_204628
	Reverse	5′-CCCTCACGGTCTTCTCCA-3′	
TNF-α	Forward	5′-AGATGGGAAGGGAATGAACC-3′	NM_204267
	Reverse	5′-GAACTGGGCGGTCATAGAA-3′	
IFN-γ	Forward	5′-CAAGTCAAAGCCGCACATC-3′	NM_205149
	Reverse	5′-TTCACCTTCTTCACGCCATC-3′	
β-actin	Forward	5′-TTACCAACACCCACACCC-3′	L08165
	Reverse	5′-ACACCTTCACCATTCCAGTT-3′	

IL-1β, interleukin one beta; IL-6, interleukin six; TNF-α, tumor necrosis factor alpha; IFN-γ, interferon gamma.

**Table 3:** Body weights and feed intake of the layers fed different level of deoxynivalenol contaminated corn DDGS

Items	Treatment					SEM	p-value1)	
	
	DDGS00	DDGS05	DDGS10	DDGS15	DDGS20		Lin	Quad
Body weight (g)								
Initial	1,849.3	1,919.5	1,916.9	1,842.1	1,915.0	12.59	0.525	0.507
Wk 2	1,842.6	1,900.4	1,900.0	1,857.8	1,893.9	11.11	0.446	0.361
Wk 4	1,956.9	2,019.7	2,006.9	1,969.1	2,015.2	12.73	0.467	0.589
Wk 6	1,987.0	2,042.0	2,008.2	1,952.0	2,028.5	11.15	0.942	0.817
Wk 8	1,993.6	2,045.9	2,022.7	1,970.9	2,032.6	11.62	0.970	0.918
Daily feed intake (g)								
Wk 2	95.5	103.2	105.2	100.3	101.1	1.20	0.312	0.085
Wk 4	126.8	128.6	129.2	131.4	128.8	0.61	0.122	0.162
Wk 6	117.8	125.3	124.9	134.0	129.0	2.28	0.067	0.411
Wk 8	111.0^a^	125.1^ab^	126.0^ab^	128.1^b^	124.0^ab^	1.96	0.028	0.025

Values are least square means of 8 replicates.

DDGS, distiller’s dried grains with solubles; SEM, pooled standard error of mean; DON, deoxynivalenol.

1) Orthogonal polynomial contrast coefficients were used to determine linear (Lin) and quadratic (Quad) effects of increasing concentrations of corn DDGS naturally contaminated with DON into layer diet.

a,b Means in the same row with different superscripts differ (p<0.05).

**Table 4:** Egg production and feed efficiency of the layers fed different level of deoxynivalenol contaminated corn DDGS

Items	Treatment					SEM	p-value1)	
	
	DDGS00	DDGS05	DDGS10	DDGS15	DDGS20		Lin	Quad
Hen day egg production (%)								
wk 2	54.69	64.29	72.13	53.57	69.87	3.762	0.462	0.679
wk 4	82.74	87.75	95.41	88.52	88.39	1.493	0.242	0.111
wk 6	86.61	93.75	90.48	91.27	88.50	1.658	0.915	0.285
wk 8	87.76	94.94	89.46	91.67	88.99	1.517	0.942	0.365
Egg mass (g/d/hen)								
wk 2	33.81	39.31	45.65	31.49	43.06	2.392	0.462	0.612
wk 4	52.26	54.98	58.79	54.98	54.73	0.890	0.433	0.076
wk 6	55.12	59.91	60.32	61.13	59.38	1.204	0.640	0.924
wk 8	48.69	60.59	56.72	57.80	56.79	1.702	0.921	0.370
Feed efficiency (g feed/g egg mass)								
wk 2	2.64	2.77	2.96	3.08	2.41	0.167	0.902	0.216
wk 4	2.44	2.38	2.19	2.40	2.38	0.038	0.694	0.136
wk 6	2.16	2.13	2.08	2.20	2.21	0.048	0.876	0.860
wk 8	2.00	2.07	2.30	2.25	2.23	0.705	0.105	0.347

Values are least square means of 8 replicates.

DDGS, distiller’s dried grains with solubles; SEM, pooled standard error of mean; DON, deoxynivalenol.

1) Orthogonal polynomial contrast coefficients were used to determine linear (Lin) and quadratic (Quad) effects of increasing concentrations of corn DDGS naturally contaminated with DON into layer diet.

**Table 5:** Egg quality indices of the layers fed different level of deoxynivalenol contaminated corn DDGS

Items	Treatment					SEM	p-value1)	
	
	DDGS00	DDGS05	DDGS10	DDGS15	DDGS20		Lin	Quad
wk 2								
Egg weight (g)	55.33	62.15	63.36	62.64	61.86	1.640	0.255	0.225
Egg width (cm)	4.45	4.37	4.44	4.38	4.47	0.020	0.337	0.358
Egg length (cm)	5.74	5.76	5.78	5.83	5.73	0.026	0.712	0.723
Shape index	77.43	75.88	76.88	75.43	78.00	0.460	0.263	0.382
Shell strength (g)	5,398.57	5,487.38	4,790.63	5,236.80	4,975.14	139.347	0.501	0.349
Shell colour (%)	25.29	26.63	29.00	29.86	28.38	0.710	0.578	0.786
Albumen height (mm)	10.43	9.00	9.13	9.14	9.00	0.200	0.260	0.408
Haugh unit	100.00	93.00	94.50	93.86	92.75	0.925	0.236	0.266
Yolk colour	5.43	5.75	5.88	6.14	5.43	0.153	0.432	0.693
wk 4								
Egg weight (g)	62.06	62.64	62.25	62.81	62.28	0.336	0.811	0.667
Egg width (cm)	4.37	4.41	4.39	4.45	4.43	0.017	0.185	0.895
Egg length (cm)	5.67	5.78	5.83	5.77	5.73	0.030	0.665	0.120
Shape index	77.09	76.28	75.40	77.07	77.51	0.454	0.619	0.201
Shell strength (g)	5,427.75	4,495.86	5,228.29	5,248.88	5,604.63	140.897	0.117	0.184
Shell colour (%)	26.57	25.00	27.13	27.63	27.75	0.584	0.704	0.527
Albumen height (mm)	9.43	9.75	9.38	10.75	10.13	0.207	0.317	0.176
Haugh unit	95.71	99.00	95.00	100.38	99.13	0.841	0.214	0.110
Yolk colour	6.75	6.75	6.88	6.88	6.63	0.116	0.885	0.542
wk 6								
Egg weight (g)	63.76	63.73	63.92	65.32	63.79	0.480	0.642	0.667
Egg width (cm)	4.42	4.44	4.47	4.52	4.37	0.019	0.069	0.226
Egg length (cm)	5.64	5.79	5.76	5.79	5.65	0.029	0.149	0.690
Shape index	78.43	76.81	77.54	78.14	77.43	0.435	0.677	0.514
Shell strength (g)	4,934.77	5,326.07	4,673.31	5,150.01	5,373.34	123.667	0.288	0.209
Shell colour (%)	27.49	28.25	32.50	28.14	28.86	0.827	0.209	0.237
Albumen height (mm)	10.67	10.83	10.54	11.89	10.37	0.205	0.093	0.061
Haugh unit	100.75	101.24	100.10	105.76	100.01	0.921	0.161	0.098
Yolk colour	6.71	5.88	5.63	7.00	6.71	0.191	0.066	0.110
wk 8								
Egg weight (g)	62.42	63.83	63.55	63.16	63.66	0.446	0.584	0.621
Egg width (cm)	4.40	4.42	4.38	4.41	4.45	0.018	0.765	0.737
Egg length (cm)	5.77	5.76	5.70	5.77	5.63	0.024	0.434	0.327
Shape index	76.20	76.63	76.77	76.42	79.04	0.356	0.244	0.329
Shell strength (g)	5,036.07	5,231.10	4,733.56	4,766.14	4,698.26	165.511	0.907	0.762
Shell colour (%)	31.00	30.43	30.50	30.25	34.50	0.752	0.402	0.701
Albumen height (mm)	9.50	10.50	10.74	9.60	9.76	0.331	0.556	0.602
Haugh unit	98.01	99.63	102.87	96.60	100.24	1.273	0.467	0.312
Yolk colour	5.00	5.29	5.50	5.38	5.63	0.189	0.970	0.915

Values are least square means of 8 replicates.

DDGS, distiller’s dried grains with solubles; SEM, pooled standard error of mean; DON, deoxynivalenol.

1) Orthogonal polynomial contrast coefficients were used to determine linear (Lin) and quadratic (Quad) effects of increasing concentrations of corn DDGS naturally contaminated with DON into layer diet.

**Table 6:** Viscera organ weights of the layers fed different level of deoxynivalenol contaminated corn DDGS

Items	Treatment					SEM	p-value1)	
	
	DDGS00	DDGS05	DDGS10	DDGS15	DDGS20		Lin	Quad
wk 4								
Liver (%)2)	2.51	2.41	2.59	2.49	2.37	0.045	0.513	0.414
Spleen (%)	0.10	0.10	0.11	0.10	0.10	0.004	0.752	0.382
Ovary (%)	2.56	2.11	2.37	2.33	2.46	0.069	0.961	0.136
Kidney (%)	0.28	0.26	0.27	0.31	0.30	0.008	0.127	0.324
wk 8								
Liver (%)	2.14	2.19	2.13	2.11	2.18	0.055	0.995	0.877
Spleen (%)	0.10	0.10	0.11	0.11	0.11	0.003	0.150	0.657
Ovary (%)	2.75	2.53	2.37	2.55	2.57	0.066	0.463	0.150
Kidney (%)	0.25	0.25	0.26	0.25	0.25	0.008	0.847	0.697

Values are least square means of 8 replicates.

DDGS, distiller’s dried grains with solubles; SEM, pooled standard error of mean; DON, deoxynivalenol.

1) Orthogonal polynomial contrast coefficients were used to determine linear (Lin) and quadratic (Quad) effects of increasing concentrations of corn DDGS naturally contaminated with DON into layer diet.

2) Organ weights as a percentage of full body weight.

**Table 7:** Blood metabolites of the layers fed different level of deoxynivalenol contaminated corn DDGS

Items	Treatment					SEM	p-value1)	
	
	DDGS00	DDGS05	DDGS10	DDGS15	DDGS20		Lin	Quad
wk 4								
Albumin (g/dL)	1.72	1.57	1.52	1.68	1.71	0.03	0.652	0.332
ALT (U/L)	2.06	1.78	2.05	1.94	1.88	0.13	0.824	0.956
AST (U/L)	177.16	165.28	161.96	170.99	167.95	1.94	0.347	0.076
BUN (mg/dL)	0.59	0.74	0.50	0.74	0.67	0.04	0.579	0.919
GGT (U/L)	27.23	27.08	28.93	29.34	26.65	0.80	0.851	0.356
Total protein (g/dL)	6.52	6.38	6.25	6.44	6.56	0.11	0.859	0.405
Globulin (g/dL)	4.80	4.77	4.79	4.76	4.85	0.09	0.883	0.814
wk 8								
Albumin (g/dL)	1.38	1.52	1.45	1.52	1.39	0.03	0.914	0.065
ALT (U/L)	0.89	0.81	0.79	0.88	0.70	0.07	0.546	0.886
AST (U/L)	167.62	175.29	167.54	160.20	165.31	3.56	0.460	0.880
BUN (mg/dL)	0.65	0.66	0.62	0.56	0.55	0.03	0.121	0.775
GGT (U/L)	21.21	26.47	26.79	26.95	25.14	0.80	0.133	0.161
Total protein (g/dL)	5.61	6.14	6.24	6.31	5.82	0.11	0.471	0.884
Globulin (g/dL)	4.24	4.62	4.78	4.80	4.43	0.10	0.419	0.252

Values are least square means of 8 replicates.

DDGS, distiller’s dried grains with solubles; SEM, pooled standard error of mean; ALT, alanine transaminase; AST, aspartate aminotransferase; BUN, blood urea nitrogen; GGT, gamma-glutamyl transferase; DON, deoxynivalenol.

1) Orthogonal polynomial contrast coefficients were used to determine linear (Lin) and quadratic (Quad) effects of increasing concentrations of corn DDGS naturally contaminated with DON into layer diet.

**Table 8:** Intestinal architecture of the layers fed different level of deoxynivalenol contaminated corn DDGS

Items	Treatment					SEM	p-value1)	
	
	DDGS00	DDGS05	DDGS10	DDGS15	DDGS20		Lin	Quad
Wk 4								
Villus height (μm)	594.36	641.25	607.20	623.92	588.97	21.034	0.862	0.549
Villus width (μm)	76.37	70.01	73.63	74.21	76.33	1.299	0.670	0.221
Crypt depth (μm)	50.33^a^	66.71^ab^	89.84^b^	91.43^b^	88.72^b^	4.453	0.001	0.076
V:C ratio	12.47^c^	10.06^b^	7.20^a^	7.16^a^	6.93^a^	0.394	0.001	0.001
Wk 8								
Villus height (μm)	747.23	832.79	700.07	723.61	737.49	24.596	0.468	0.951
Villus width (μm)	76.22	80.77	85.22	74.07	85.96	2.205	0.409	0.960
Crypt depth (μm)	69.13^a^	100.03^ab^	110.47^ab^^c^	126.34^b^^c^	152.78^c^	7.130	0.001	0.941
V:C ratio	11.31^c^	9.13^b^	6.63^a^	5.92^a^	5.15^a^	0.436	0.001	0.017

Values are least square means of 8 replicates.

DDGS, distiller’s dried grains with solubles; SEM, pooled standard error of mean; V:C, villus height to crypt depth ratio; DON, deoxynivalenol.

1) Orthogonal polynomial contrast coefficients were used to determine linear (Lin) and quadratic (Quad) effects of increasing concentrations of corn DDGS naturally contaminated with DON into layer diet.

a,b Means in the same row with different superscripts differ (p<0.05).

**Table 9:** Cytokine expression of the layers fed different level of deoxynivalenol contaminated corn DDGS

Items	Treatment					SEM	p value1)	
	
	DDGS00	DDGS05	DDGS10	DDGS15	DDGS20		Lin	Quad
Wk 4								
IL-1β	2.34	1.54	58.74	21.46	95.76	15.204	0.087	0.678
IL-6	4.11	2.35	37.02	22.30	70.67	11.708	0.113	0.639
IFN-γ	58.61	41.24	1,210.21	886.47	2,230.95	415.566	0.140	0.757
Wk 8								
IL-1β	0.26^b^	0.25^b^	0.17^ab^	0.01^a^	0.03^a^	0.034	0.001	0.325
IL-6	0.01	0.03	0.01	0.00	0.01	0.003	0.337	0.888
IFN-γ	0.01	0.01	0.01	0.00	0.00	0.002	0.084	0.519
TNF-α	0.27	0.28	0.30	0.12	0.23	0.026	0.209	0.991

Values are least square means of 8 replicates.

DDGS, distiller’s dried grains with solubles; SEM, pooled standard error of mean; IL-1β, interleukin one beta; IL-6, interleukin six; TNF-α, tumor necrosis factor alpha; IFN-γ, interferon gamma; DON, deoxynivalenol.

1) Orthogonal polynomial contrast coefficients were used to determine linear (Lin) and quadratic (Quad) effects of increasing concentrations of corn DDGS naturally contaminated with DON into layer diet.

a,b Means in the same row with different superscripts differ (p<0.05).
